# Gut Microbiota and Metabolites Mediate Health Benefits of Oat and Oat Bran Consumption in IBD Mice

**DOI:** 10.3390/nu16244365

**Published:** 2024-12-18

**Authors:** Wen Duan, Bisheng Zheng, Tong Li, Ruihai Liu

**Affiliations:** 1 School of Food Sciences and Engineering, South China University of Technology, Guangzhou 510641, China; 15754367187@163.com (W.D.); febzheng@scut.edu.cn (B.Z.); 2 Overseas Expertise Introduction Center for Discipline Innovation of Food Nutrition and Human Health, School of Food Sciences and Engineering, South China University of Technology, Guangzhou 510641, China; 3 Department of Food Science, Cornell University, Ithaca, NY 14853, USA; tl24@cornell.edu

**Keywords:** oats, bran, inflammatory bowel disease, microbiota, whole grain

## Abstract

Background/Objectives: Inflammatory bowel disease (IBD) is a chronic condition influenced by a variety of factors, including genetics, the environment, and gut microbiota. The incidence of IBD is increasing globally. Previous studies have shown that interactions between diet and gut microbiota influence the pathogenesis and treatment of IBD. Proper dietary nutrition including oat and oat bran regulates chronic inflammation, which is essential for individual health, and is one of the essential factors in reducing inflammation in the body and keeping the immune system functioning properly, which plays a role in the prevention and treatment of diseases. However, the mechanism of action of whether oat and oat bran will alleviate chronic inflammation by modulating intestinal flora and metabolites remains unknown. Methods: Therefore, in this study, we have used a mouse model of dextran sulfate sodium (DSS) chronic colitis to analyze the composition of intestinal microbiota, short-chain fatty acid content, and the expression of the relevant genes. Results: The results showed that diets supplemented with oat and oat bran improved intestinal barrier parameters, decreased the levels of inflammatory factors, modulated the composition of intestinal microbiota, and increased the content of short-chain fatty acids. Conclusions: This study provides strong evidence that dietary interventions with oats or oat bran may have potential applications in clinical nutrition and dietary interventions for chronic IBD.

## 1. Introduction

Inflammation is thought to have a central role in many chronic diseases. Abnormal inflammation production can even compromise the immune system, which in turn mediates disturbances in tissue homeostasis and causes chronic damage to the organism [1]. Chronic inflammation is a nonspecific, persistent state of low-grade inflammation presented by the organism in response to prolonged, low-dose stimulation by specific immunogens. Chronic inflammatory diseases are on the rise worldwide. There is evidence that dysbiosis of the gut flora is a hallmark of various chronic diseases [2]. IBD is a chronic progressive disease that belongs to the group of gastrointestinal disorders with a more complex etiology [3]. The progression to cancer in IBD is primarily linked to repeated cycles of inflammation and repair of the intestinal mucosa. This condition may cause symptoms including weight reduction, abdominal pain, loose bowel movements, and fecal blood. The influence of the microbiota on IBD has been studied for many years, but a direct causal relationship has not been established. Dysregulation of the gut microbiota can alter host–microbe interactions and affect the host immune system and nutrient absorption and metabolism, which in turn disrupts the intestinal epithelial mucosal barrier and promotes intestinal inflammation. A growing body of compelling evidence supports the role of gut microbes in regulating IBD [3]. Current clinical treatments for colitis typically involve the use of immunosuppressants, antibiotics, and other medications to reduce intestinal inflammation. It has also been shown that diet may have anti-inflammatory benefits through gut microbiota-derived bioactive substances that act as inhibitors of inflammatory pathways [4].

The human body metabolizes its daily dietary intake in the presence of intestinal flora, and the dietary structure exerts a notable influence on the composition and distribution of microbiota. Diet can influence the course of IBD by altering the gut microbiome and significantly affecting the intestinal mucosal barrier, thereby modulating host immunity [5]. It has been shown that diet can provide a rich source of bioactive compounds, both directly and indirectly (through the gut microbiota), which have local and systemic effects on immune function and mediate the development of inflammatory responses [4]. Oats are a traditional cereal grain considered to be the best among whole grains in terms of whole-value nutrients and are a healthy source of dietary fiber, protein, starch, lipids, polyphenols, vitamins, and minerals. They promote the proliferation of beneficial bacteria and reduce harmful bacteria, protecting the host’s health [6]. Studies have shown that a diet supplemented with oats can more effectively alleviate disease in mice and inhibit obesity-induced intestinal barrier dysfunction [7]. Oat bran, a by-product of oat processing, is also nutrient-rich, containing protein, vitamins, minerals, and a high amount of dietary fiber. However, long-term, single-nutrient intake of oat bran can lead to nutritional imbalances. Furthermore, most oat bran is currently used as animal feed, resulting in low utilization and limited economic value [8]. Kristek et al. (2019) conducted an in vitro digestive fermentation test using normal human feces and found that oat bran decreases the diversity of intestinal microbial populations and also has a bifidogenic effect, which enhances Bacteroides and Enterobacter, and that a higher dose of oat bran produces higher amounts of acetic and propionic acids. However, it reduces butyric acid production [9]. Dietary fiber constitutes approximately 20% of the composition of oats and is predominantly sourced from oat bran. Singh et al. (2018) discovered that a diet abundant in soluble dietary fiber components such as inulin, pectin, and oligofructose did not elicit substantial variations in the relative abundance of gut microbiota and thick-walled phyla among congenitally immune-deficient mice. However, it did lead to a notable decrease in the diversity and variety of gut microbial species. The underlying cause of this significant reduction is attributed to microbial transmissibility [10]. Notably, the primary prebiotic in oat bran, β-glucan, is a well-recognized bioactive compound known to lower blood glucose and lipid levels while promoting gut health [8,11]. Furthermore, oat bran is abundant in phenolic acids, compounds with potent anti-inflammatory properties. Recent studies suggest that phenolic acids also serve as potential prebiotics, capable of enhancing the gut microbiota’s composition and structure, which positively influences overall health [12]. Oat phenolics, a class of secondary metabolites, are regarded as the most potent antioxidants in oats, capable of effectively neutralizing excess free radicals and preventing chronic diseases induced by oxidative stress. Data from our laboratory revealed that the total phenolic content in oats and oat bran is 123.12 ± 7.3 mg GAE/100 g and 164.35 ± 1.2 mg GAE/100 g, respectively. Among these, ferulic acid, caffeic acid, and coumaric acid were identified as the predominant phenolic acids. The phenolic acids in oats exhibit structural diversity, with free phenolic acids primarily concentrated in the hulls, while bound phenolic acids are attached to the grain cell walls via ester bonds, requiring enzymatic hydrolysis to be released [13]. Zhao et al. found that incorporating polyphenol-rich grape flour into the diet reduced the incidence of inflammatory colon cancer in mice by 29% compared to a normal diet, and that this supplemental intake of grape flour increased the relative abundance of some butyrate-producing bacteria, which in turn increased fecal butyrate levels [14]. However, the effect of oats and oat bran on chronic intestinal disease in mice is unknown, as is the mechanism of action as to whether oats and oat bran alleviate chronic colitis in mice by modulating gut microbiota and metabolism.

Therefore, the objective of this study was to study the effects of oats and oat bran on the composition of intestinal microbiota and fecal metabolites in mice with DSS-induced chronic colitis. The final aim was to reveal some beneficial mechanisms of action of oats and oat bran, thus providing a basis for their comprehensive utilization.

## 2. Materials and Methods

### 2.1. Materials and Reagents

Oats and oat bran were sourced from Yangu Fang Company in Wuchuan, Inner Mongolia, China. DSS (40000 Da, MP Biomedicals, Inc., Solon, OH, USA), aspartate aminotransferase (AST), and alanine aminotransferase (ALT) enzymes were sourced from the Nanjing Jiancheng Institute of Bioengineering (Nanjing, Jiangsu, China). We acquired the RNA reverse transcription kit and real-time fluorescence quantitative PCR kit from Sangon Biotech Co., Ltd. (Shanghai, China).

### 2.2. Animals and Experimental Design

**Figure 1:**
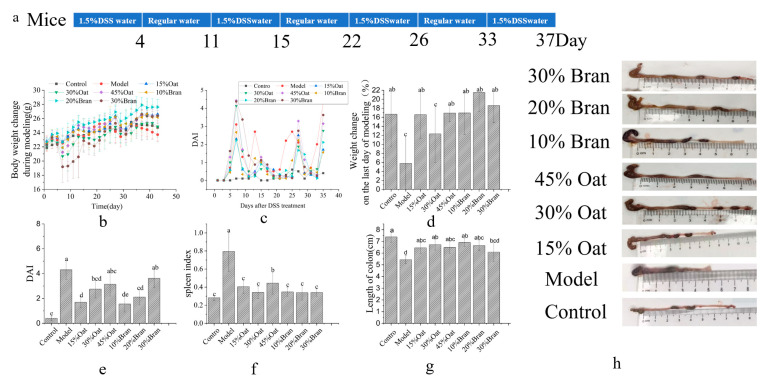
Effects of oat and bran on mice with DSS-induced colitis. (**a**) Schematic diagram of the constructed model of DSS-induced chronic colitis (*n* = 10 per group). (**b**) Weight change during modeling. (**c**) DAI score. (**d**) Weight change on the last day of modeling. (**e**) DAI score. (**f**) Spleen index. (**g**) Colon length. (**h**) Images depicting the colons of the mice. Different lowercase represent significant differences among groups.

Male C57BL/6j mice (SPF, 8 weeks, 20 g ± 2 g) were bought from Guangdong Sijia Jingda Biological Technology Co., Ltd. (Guangzhou, China). The animal experiments conducted adhered to the Guide for the Care and Use of Laboratory Animals, approved by the ethics review board of South China Agricultural University (SCAU-2022B201), in accordance with the National Research Council’s guidelines. The experimental procedure followed the requirements of the ARRIVE guidelines. All mice (80) were fed ad libitum during the experimental period, housed in an environment with a temperature of 25 ± 2 °C, and exposed to light and dark (12 h/12 h) alternately. At the end of one week of acclimatization, all mice were randomly divided into the control group, model group, 15%, 30%, and 45% oat dose groups, and 10% oat bran, 20% oat bran, and 30% bran dose groups, with a total of 10 mice in each group. Each group of mice was housed individually in separate cages. The dietary ingredients and nutrient compositions are shown in Table S1. Except for the control group, mice were subjected to four cycles of 1.5% DSS in their drinking water, where each cycle consisted of 4 days of DSS-induced colonic inflammation followed by 7 days of recovery with normal water. The specific model construction method is shown in Figure 1a. On the last day before sacrifice, all mice were fasted and stool samples were gathered and kept in a −80 °C refrigerator. On the day of sacrifice, the mice’s final body weights were noted, and blood samples were obtained from their hearts, allowed to clot at 4 °C, centrifuged, and the serum was stored in an ultra-low temperature refrigerator. After dissecting the mice, the colon length was assessed immediately, and a suitable amount of the colon was fixed in 4% formalin.

### 2.3. Mouse Biochemical Parameters

Disease activity index (DAI) in mice was evaluated with reference to the human criteria in Ito et al. [15]. DAI in mice was determined by weight loss (0 = no loss, 1 = 1–5% loss, 2 = 6–10% loss, and 3 = 11–20% loss), stool consistency (0 = normal, 1 = soft with some hardness, 2 = soft and moist, and 3 = liquid diarrhea), and the presence of blood in feces (0 = absent, 1 = trace, 2 = present, and 3 = abundant). AST and ALT activities were measured by commercial kits. TNF-α, IL-6, and IL-10 were measured in colon samples using enzyme-linked immunosorbent assay (Elisa) kits (Shenzhen NeoBioscience Biotechnology Co., Ltd., Shenzhen, China).

### 2.4. Histopathological Analysis

The fixed colon was first dehydrated, paraffin-embedded, sectioned, and dewaxed. The above sections were then stained with hematoxylin. Subsequently, they were sequentially stained with differentiation and rebluing solutions for differentiation and reblue treatment. Then, dehydration and eosin staining were performed. Finally, the sections were dehydrated and sealed again, then observed under the microscope before images were collected [16].

### 2.5. Analysis of Short-Chain Fatty Acids

Short-chain fatty acids were analyzed according to previously published methods [17]. Fecal samples were quickly added (500 μL) to 0.5% phosphoric acid solution (content mass: phosphoric acid solution = 1:10), vortexed thoroughly for 5 min, and centrifuged at a speed of 15,000× *g* for a duration of 10 min at a temperature of 4 °C. An equal volume of ethyl acetate was added and the solution was vortexed for 5 min, followed by another centrifugation process (15,000× *g*, 10 min). This was followed by gas chromatography–mass spectrometry (GC–MS) analysis. Chromatographic column: polar capillary column DB-WAX, helium carrier gas concentration 1 m L/min. The feeding procedure was similar to the method of Li et al. [18].

### 2.6. Gut Microbiota Composition Analysis

The fecal microbial community’s total DNA was isolated using a commercial kit (Omega Bio-tek, Qiagen, Germantown, MY, USA) [19]. The quality, concentration, and purity of RNA were determined using a micronucleic acid protein analyzer(Thermo Fisher, Waltham, MA, USA). The 16S rDNA gene spanning the V1–V9 region was selected with forward primer 27F5′-AGRGTTYGATYMTGGGCTCAG-3′ and reverse primer 1492R5′-RGYTACCTTGTTACGACTT-3′. The PCR products were recovered, purified by 2% agarose gel electrophoresis, and quantified. Library construction of amplified fragments was performed using Illumina platform sequencing protocols and high-throughput sequencing was performed using a NovaSeq 6000 sequencer(Illumina, San Diego, CA, USA). In order to obtain the taxonomic information of the species corresponding to each OTU, the samples were processed using the Usearch software(version 10). The uclust algorithm was used to taxonomically analyze the representative sequences of OTUs with a 98.65% similarity level. Utilizing the outcomes from the OTU analyses, the alpha and beta diversity of fecal matter was analyzed using mothur, and the community structure was statistically analyzed at each taxonomic level. All graphics were processed at the OmicStudio online website (http://www.cloud.biomicroclass.com/CloudPlatform)(accessed on 2 June 2023).

### 2.7. Gene Expression Analysis

Colon tissue samples were placed in a centrifuge tube, and a lysis solution, along with five grinding beads, was added. The tissues were then homogenized using a high-throughput tissue crusher(Shanghai Wanbai Biotechnology Co., Shanghai, China). The supernatant was separated by centrifugation, and 200 μL of chloroform was added to the tube. Next, it was vortexed for 15 s and centrifuged again to collect the upper layer of the supernatant. Next, 0.5 mL of isopropanol was added, and the supernatant was centrifuged after 10 min. This was followed by the addition of 75% ethanol, and the supernatant was removed by centrifugation after shaking and mixing. Finally, after drying for a duration of 10 min at room temperature, 50 μL of enzyme-free water was added. The RNA concentration and purity were analyzed by a micronucleic acid protein analyzer and then stored in a −80 °C refrigerator for future use as a backup [20]. Primer sequences were as follows: GAPDH, (F: CTCGTCCCGTAGACAAAATGGT, R: GAGGTCAATGAAGGGGTCGTT); TNF-α, (F: CAGGCGGTGCCTATGTCTC, R: CTCGTCCCGTAGACAAAATGGT); IL-10, (F: TGAATTCCCTGGGTGAGAAGC, R: CACCTTGGTCTTGGAGCTTATT); Claudin-1,(F: GGGTTTCATCCTGGCTTCT, R: GTATCTGCCCGGTGCTTT); Claudin-5, (F: GCCTTCCTGGACCACAACA, R: GAGTGCTACCCGTGCCTTAA); IL-6, (F: CTGCAAGAGACTTCCATCCAG, R: AGTGGTATAGACAGGTCTGTTGG).

### 2.8. Statistical Analysis

The data were reported as mean ± SD. Statistical analyses included one-way ANOVA and Duncan’s multiple range test, performed using SPSS 21.0 (SPSS Inc., Chicago, IL, USA). To assess bacterial abundance variations between groups, the Wilcoxon rank sum test was applied, while significant differences were further characterized by the Kruskal–Wallis (KW) non-parametric test. The alpha diversity index was calculated using the R language(version 4.0.2) (Chao1, ACE, Shannon, Simpson). *p* < 0.05 was considered statistically significant.

## 3. Results

### 3.1. Oats and Oat Bran Improved the Symptoms of Colitis in Mice

To further investigate the effects of oats and bran on intestinal inflammation, a mouse model of chronic colitis was established through the induction of DSS in this research. Studies have shown that colitis is often accompanied by weight loss. As shown in Figure 1b, after the modeling period, there was a slight increase in the weight of the mice in each group. Compared with the model group, weight gain was observed in groups fed with oats and bran, while the model group had the most pronounced weight loss (Figure 1d).

DAI scores were significantly elevated in mice at each recovery cycle after being fed DSS water, as well as being significantly elevated in the model group compared to the control group. However, they did not differ significantly from the group receiving 45% oats and 30% oat bran, whereas the low-to-medium dose groups of oat and oat bran had significantly reduced DAI scores (Figure 1c,e). The colon length of mice in the model group (5.43 ± 0.34 cm) was significantly shorter compared to that of the mice in the control group (7.38 ± 0.45 cm) (Figure 1g). Compared with the model group, colon length was significantly increased after feeding oats to low- and medium–high-dose groups and bran to 10% and 20% dose groups, whereas there was no significant difference in colon length between the model group and the mice in high-dose bran groups. As shown in Figure 1f, when compared to the control group, the spleen of the model group was enlarged and the spleen index was significantly increased. Compared with the model group, the spleen index of the rest of the groups were decreased.

### 3.2. Oat and Oat Bran Ameliorate Histologic Damage to the Colon in Colon Colitis

**Figure 2:**
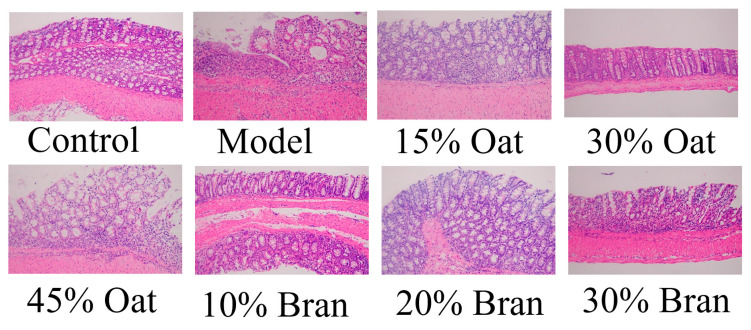
Effects of oats and bran on colon tissue in mice (*n* = 10 per group). Representative H&E staining (50× magnification).

To investigate the effects of oatmeal and bran on the physiological characteristics of the colon, HE staining was used to assess the colonic tissues of mice. In Figure 2, the colonic tissues of control mice showed intact mucosa, deep crypts, abundant cup cells, clear muscularis propria structures, no inflammation-associated cells, and normal pathologic features. Mice in the model group showed obvious changes in pathological features, with the disappearance of crypts, a massive reduction in cup cells, massive inflammatory cell infiltration, and submucosal edema. A mild inflammatory infiltration was noted in the epithelial mucosa of the colon in the 45% oat group and the 30% bran group. The degree of infiltration of inflammatory cells was higher and damage to the intestinal wall was still visible, although the rest of them did not have any obvious abnormality. The structural integrity of the colonic mucosa of the mice in the 15% oat group, the 30% oat group, the 10% bran group, and the 20% bran group was reduced by a decrease in pathological damage and a decrease in histological scores, which effectively improved the integrity of the colonic tissues. The above colitis symptoms were alleviated to different degrees in the different oat and bran groups, which suggests that oats and bran can inhibit DSS-induced colonic damage and inflammatory responses.

### 3.3. Oat and Oat Bran Improve Expression of Inflammatory Factors

**Figure 3:**
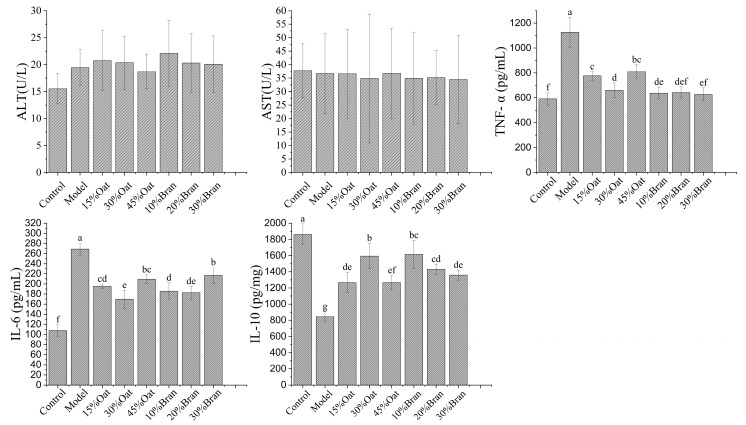
Effects of oat and bran on levels of AST, ALT, TNF-α, IL-6, and IL-10 in mice (*n* = 10 per group). Different lowercase represent significant differences among groups.

As can be seen in Figure 3, the extent of hepatocyte damage in the oat and bran intervention groups was comparable to that observed in the model control group, indicating that these interventions were non-toxic. To further evaluate the secretion of inflammation-related cytokines, the secreted cytokines were detected in the serum of different groups of mice using Elisa assays. In Figure 3, compared to the control group, the colonic tissues of mice in the model group exhibited significantly elevated levels of IL-6 and TNF-α, as well as a significant decrease in IL-10. Oat and bran intervention groups all significantly reduced the secretion levels of the inflammatory factors IL-6 and TNF-α and increased the secretion of the anti-inflammatory factor IL-10 in the colonic tissues of mice, with the 30% oat, 10% bran, and 20% bran groups having the best effect in inhibiting the secretion of inflammatory factor levels.

### 3.4. Effect of Oat and Oat Bran on the Content of SCFAs in Mouse Colon Tissue

**Figure 4:**
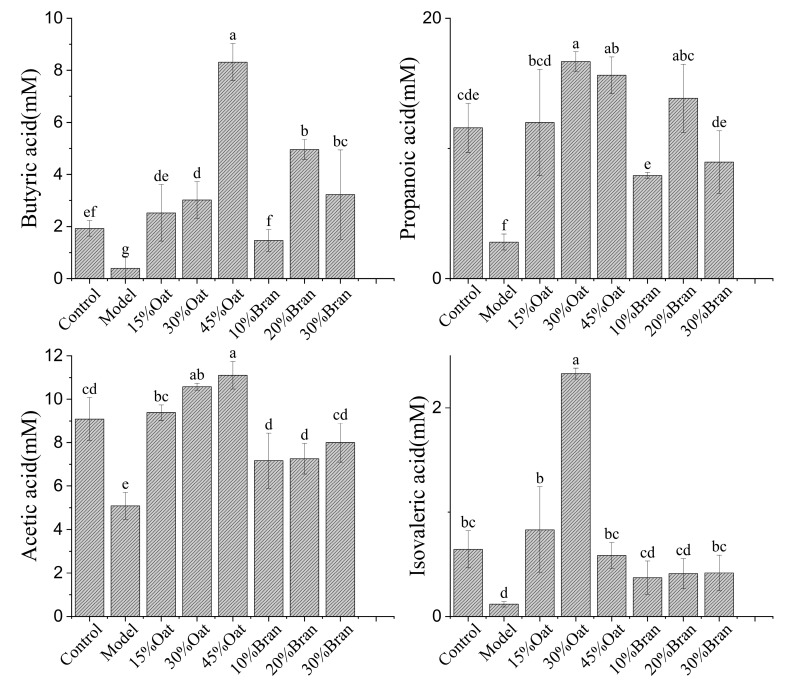
SCFA content in oat and oat bran. Butyric, propionic, acetic, and isovaleric acids (*n* = 10 per group). Different lowercase represent significant differences among groups.

In Figure 4, the levels of acetic acid, propionic acid, butyric acid, and isovaleric acid in the feces of mice in the model group were 1.31 ± 0.25 mM, 1.31 ± 0.25 mM, 0.27 ± 0.05 mM, and 0.29 ± 0.04 mM, respectively, which were significantly lower compared to those in the control group. The oat and oat bran groups significantly inhibited the decrease in SCFA content induced by DSS. Among them, the 45% oats group, 20% bran group, and 30% bran group significantly elevated butyric acid levels in mice with colitis, and the 30% oats group and 20% bran group significantly increased the content of propionic acid in colitis mice. The 30% oats group and 45% oats group significantly boosted acetic acid levels in colitis mice and the 30% oats group increased the content of isovaleric acid in colitis mice. In addition, the levels of butyric acid, propionic acid, acetic acid, and isovaleric acid were lower in all oat bran groups, with the exception of butyric acid and propionic acid in the 20% bran group, which were higher than in the control group.

### 3.5. Effect of Oat and Oat Bran on Gut Microbiota

**Figure 5:**
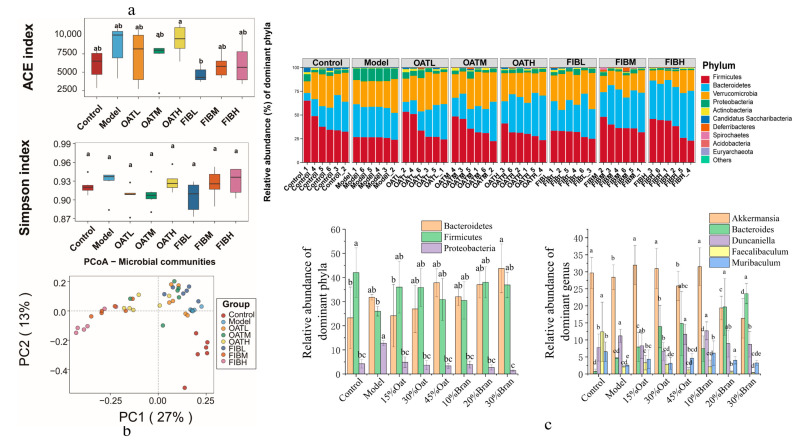
Fecal microbiota in mice (*n* = 10 per group). (**a**) ACE and Simpson index of alpha diversity. (**b**) PCoA based on Weighted UniFrac distances. (**c**) The abundance of bacteria at the phylum level. Different lowercase represent significant differences among groups.

**Figure 6:**
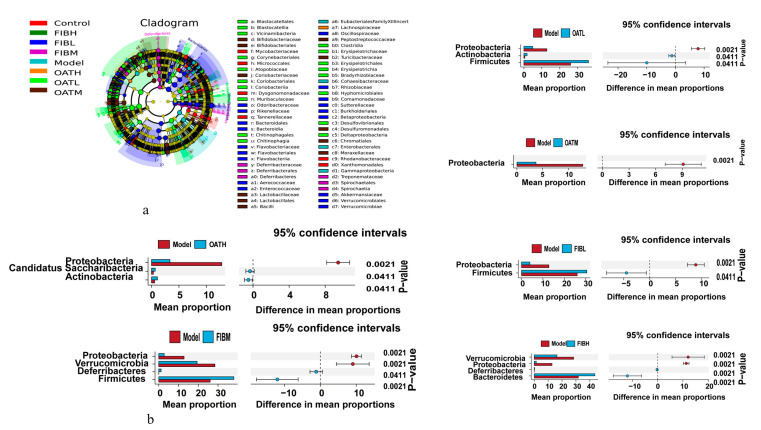
Fecal microbiota in mice (*n* = 10 per group). (**a**) The cladogram depicting taxa from the two groups (**b**), along with LDA scores, was identified through Linear Discriminant Analysis Effect Size (LEfSe) analysis, with a cutoff set at an absolute log10 LDA score > 7.0.

To further investigate whether oat and oat bran interventions in colitis regulate the intestinal flora by altering its structure, changes in the structure of the microbial community in the model and experimental groups were assessed with the help of 16s rRNA sequencing. In Figure 5a, there were no significant difference between the groups in terms of ACE and Simpson’s index. This indicates that the administration of oat and oat bran did not change the number and abundance of species in the gut microbiota of mice. The PCoA results revealed a significant distinction between the control and model groups. Additionally, the oat and oat bran intervention groups were close to each other and were separated from the distribution of colonic microbial species in the DSS group (Figure 5b). The effects of oat and oat bran on the microbiota of IBD mice were investigated by observing the community structure of intestinal microorganisms at the phylum level and genus level in each group of mice. The microbiota in the samples mainly consisted of Bacteroidetes, Firmicutes, Proteobacteria, and some other lesser-occupied phyla. The increase in Proteobacteria reflects the disruption of the gut microbial community. Figure 5c showed that the abundance of fecal Proteobacteria was upregulated in IBD mice compared to control mice, whereas the abundance of Proteobacteria decreased after oat and oat bran interventions. Compared to the control group, the model group exhibited a higher abundance of Bacteroidetes and Proteobacteria. Following oat and oat bran interventions, the abundance of Bacteroidetes and Proteobacteria decreased in the low-dose oat group, medium-dose oat group, and low-dose bran group compared to the model group. Notably, the reduction in fecal Proteobacteria abundance was most pronounced in the low-dose oat group. Firmicutes abundance was notably reduced in the model group when compared to the control group. However, in both the oat and oat bran intervention groups, Firmicutes abundance was higher than in the model group, although the difference was not statistically significant. The F/B value of the model group was lower than that of the control group and was increased in both the oat and bran groups compared to the model group. In addition, at the genus level, the model group showed a significant decrease in the relative abundance of Akkermansia, Muribaculum, and Faecalibaculum, while the relative abundance of Bacteroides and Duncaniella was increased in comparison to the control group. However, compared to the model group, the relative abundance of different microbiota was altered in both the oat and oat bran groups, with the relative abundance of Bacteroides, Muribaculum, and Faecalibaculum being upregulated in the oat 15% dose group, the oat 30% dose group, and the oat bran 10% dose intervention group. In order to compare the dominant bacteria between groups and determine which bacteria may be the main microbiota responsible for the effects of oats and bran on IBD mice, a linear discriminant analysis (LDA) was performed using LEfSe analysis with the LDA threshold set at 7 (Figure 6a). The results showed that Proteobacteria and Verrucomicrobia were enriched in the intestinal tract of mice in the model group. After oat and oat bran interventions, the low-dose oat group and low-dose bran group showed enrichment of Firmicutes compared to the model group (Figure 6b).

### 3.6. Effect of Oat and Oat Bran on the Expression Level of Related Genes

**Figure 7:**
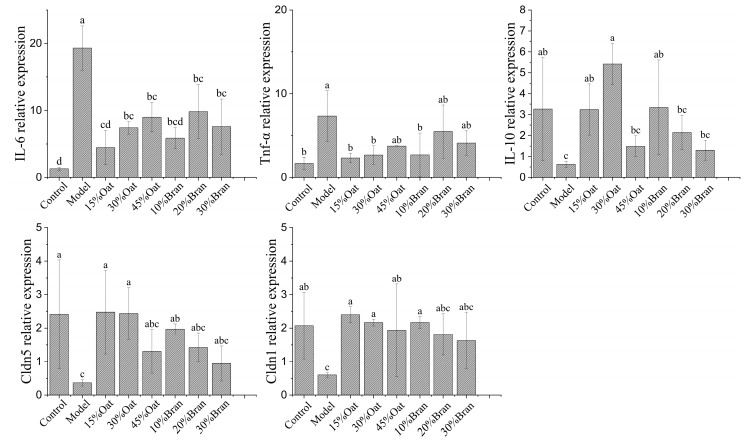
The relative mRNA expression levels of IL-6, TNF-α, IL-10, Cldn1, and Cldn5 in colon tissues (*n* = 10 per group). Different lowercase represent significant differences among groups.

In Figure 7, the gene expression levels of IL-6 and TNF-α were low in the colon of the control group. In contrast, the expression levels of IL-6 and TNF-α were significantly increased in the colon of the model group, indicating that DSS activated the relevant inflammatory signaling pathway and promoted the gene expression of IL-6 and TNF-α. Compared with the model group, different concentrations of oat and bran treatment groups could promote the expression of IL-10 and inhibit the gene expression of IL-6 and TNF-α, thus mitigating the mucosal damage caused by DSS-induced colitis. Furthermore, the oat and oat bran intervention groups were able to increase the expression levels of Claudin-1 and Claudin-5 genes in the colon of DSS-induced colitis mice. This intervention reduces the infiltration of harmful substances and associated damage, ultimately decreasing the occurrence and progression of colitis.

## 4. Discussion

Existing studies suggest that IBD is caused by the immune activation response of the body due to the disruption of the balance between gut microbiota composition and immune defects of the intestinal mucosa [21]. Dietary interventions are an important aspect of IBD management. The typical Western diet has been strongly linked to a heightened incidence of IBD, whereas the consumption of whole grains shows a significant inverse association. Previous studies have explored the mechanisms by which dietary fiber, phytochemicals, and amino acids—key beneficial constituents of grains—ameliorate IBD, revealing their potential in modulating gut microbiota, reducing inflammation, and enhancing intestinal barrier function [22,23]. In this experiment, we used a model of chronic colitis in mice by using three half-cycles of “4 days of drinking DSS and 7 days of normal drinking”. We evaluated the construction of the chronic colitis model by observing the changes in the body weight, DAI score, spleen index, and colonic microscopic score of the mice, and investigated the effects of oats and oat bran on mice with chronic colitis, along with the related mechanisms. The DAI scores of mice in the model group were significantly increased, including reductions in body mass and the presence of diarrhea and blood in the stool. Shortening of the colon is an obvious pathological symptom in IBD, and the colon length of mice in the model group was significantly shortened, and the colonic mucosa was severely edematous and necrotic, with a reduction in goblet cells and infiltration of inflammatory cells. Previous studies have indicated that mice display similar symptoms following DSS induction [18]. In contrast, mice in the oat- and oat bran-treated groups gained weight, and the examination of intestinal contents revealed a good structure of fecal pellets, a significant increase in colon length, and a better improvement in colonic histopathology. Oat and oat bran improved the clinical manifestations of DSS-induced colitis in mice. Specifically, low-dose oat and oat bran treatments were effective in alleviating colon shortening, whereas the high-concentration bran group was less effective. Both oats and bran, when administered at low-to-medium doses, reduced the incidence of diarrhea and fecal occult blood, improved macroscopic scores related to fecal and occult blood, and diminished spleen enlargement induced by DSS treatment.

Chronic intestinal inflammation is the result of an abnormal mucosal immune response. Detection of inflammatory factors in the colon is correlated to the level of inflammation in mice with colitis [24]. IL-10 acts as an immunosuppressive factor, which prevents the immune system from developing an allergic response to harmless intestinal antigens, thus avoiding intestinal inflammation. In addition, IL-10 serves as a key immune-regulating cytokine that protects the intestinal mucosal barrier by effectively suppressing the excessive immune response in the colonic mucosa triggered by inflammation [25,26]. Increased intestinal permeability leads to chronic inflammation. TNF-α and IL-6 play crucial roles in the pathophysiological process of IBD by modulating the mucosal immune system and altering epithelial integrity, which can lead to colonic damage and exacerbate tissue inflammation [27]. The results of this experimental study indicate that DSS treatment can increase intestinal permeability in mice, allowing more inflammatory factors to pass through the intestinal barrier, thereby causing an intestinal inflammatory response. Conversely, polyphenols present in oats and bran have been shown to restore the host–microbe equilibrium within the gut by modulating intestinal macrophages, suppressing T-cell activation, enhancing IL-10 expression, and markedly reducing the TNF-α/IL-6 ratio. These combined effects significantly mitigate inflammation and support intestinal health [28].

SCFAs are metabolites produced during the fermentation process of intestinal bacteria, and studies have shown that SCFA-producing bacteria in the feces of IBD patients are significantly reduced, and the concentrations of fecal SCFAs were decreased [29]. Dietary fiber is fermented by the microbiota in the cecum and colon, resulting in the production of SCFAs. A reduction in SCFA levels has been associated with the severity of IBD [30]. In this experiment, we found that the content of SCFAs in the feces of mice in the DSS model group was significantly decreased, which may be related to the dysregulation of intestinal flora in mice stimulated by DSS, resulting in a decrease in SCFA production. Li et al. (2024) similarly found that DSS intake reduced SCFA content in mouse feces, which may be due to the fact that microorganisms utilize either proteins or fats when there is an insufficient supply of fermentable fibers, resulting in the reduced fermentation activity of SCFAs as secondary end products [31]. The fact that the oat group was more effective than the bran group in alleviating the reduction of SCFAs in colitis mice may stem from the dual role of oats, as polyphenols and dietary fiber both contribute to alleviating intestinal inflammation. However, excessive dietary fiber intake could exacerbate IBD by impairing intestinal barrier integrity and disrupting immune functions, highlighting the need for balanced consumption to optimize benefits [32].

Disturbances in the microbiota are important triggers for the development of intestinal inflammation in IBD. Microbiota is related to the functional regulation of the body’s immunity and maintains a mutually beneficial relationship with the host [33]. The dysfunction of microbiota is one of the fundamental characteristics of intestinal inflammation progression, manifested by changes in the composition and abundance of bacterial communities [34]. Restoration of the microbiota through modulation has emerged as a potential strategy for the clinical management of IBD [35]. In this study, the microbiota of colitis mice showed an increase in levels of the bacteria Bacteroidetes and a decrease in the level of the bacteria Proteobacteria and Firmicutes. Feng et al. (2020) similarly observed an increase in the relative abundance of Bacteroidetes and a decrease in Firmicutes [36]. Available evidence indicates that polyphenolic compounds influence intestinal ecology through their dynamic interactions with gut microbiota. These compounds can modulate intestinal metabolites, thereby affecting bacterial growth and altering the abundance and composition of gut flora. Conversely, gut microbes metabolize polyphenols into bioactive compounds, which further influence the microbial community, creating a bidirectional regulatory relationship that sustains intestinal health [37]. Furthermore, oat polyphenols are metabolized by intestinal microorganisms, facilitating the digestion and absorption of phenolic compounds and significantly enhancing their bioavailability within the gastrointestinal tract [8]. Administration of oat and oat bran to colitis mice resulted in changes to the compositional structure of the microbiota. This effect is likely attributed to the polyphenols present in oats and bran, which enhance microbial diversity by increasing the abundance of beneficial bacteria while suppressing harmful ones. These shifts in microbial populations subsequently influence the metabolites of the gut microbiota, thereby contributing to the alleviation of IBD.

Bacteroides strains have been reported to activate inflammatory pathways through the expression of specific transporter proteins and lipoproteins to regulate the levels of inflammatory factors in vivo and influence the inflammatory response in the intestine. Muribaculum is linked to the production of SCFAs, which regulate environmental homeostasis inside the intestine [38]. Oats and bran were shown to promote the growth and multiplication of Firmicutes and Proteobacteria and inhibit the growth of Bacteroidetes. IBD is characterized by a disturbance in the microbial composition and in the balance between the microbiota and the mucosal immune response. Bacteroidaceae have been documented to be strongly associated with the onset and progression of inflammation by promoting the production of succinic acid, which causes intestinal mucosal erosions and intestinal submucosal edema. Pisani et al. (2023) found that the reduction of thick-walled bacteria also affects the development of the disease in patients with IBD. This result further supports the idea that IBD is strongly associated with intestinal dysbiosis [39]. Thus, the consumption of oat and oat bran can reduce the relative proportion of harmful bacteria linked to gut inflammation in the gut, possibly because gut microbes can utilize polyphenols and break them down into various metabolites. They can also increase the synthesis of, for example, SCFAs, which in turn protect the gut barrier. Previous studies have indicated that bioactive compounds derived from natural products can alleviate IBD by repairing the intestinal epithelial barrier and protecting tight junction proteins [40,41]. We hypothesized that oat and oat bran might have similar effects. The results indicated that oat and oat bran interventions significantly upregulated the expression of the colonic epithelial barrier genes Claudin-1 and Claudin-5. This suggests that polyphenols and dietary fiber from oat and oat bran may enhance tight junction protein secretion in inflamed conditions, thereby reducing intestinal permeability and mitigating the dysfunction of the intestinal barrier induced by DSS.

## 5. Conclusions

The incidence of IBD is on the rise globally and has attracted a great deal of attention. A crucial first step in preventing and managing many chronic diseases is maintaining a healthy diet. Our study suggests that the protective effects of oat and oat bran against chronic colitis in DSS colitis mice may be mediated by enhancing intestinal barrier function. This is achieved by preventing the downregulation of tight junction proteins Claudin-1 and Claudin-5, reducing the expression of IL-6 and TNF-α in colonic tissues, inhibiting immune cell infiltration, and modulating the gut microbiota to increase the relative abundance of beneficial bacteria such as Firmicutes and Proteobacteria while decreasing harmful bacteria such as Bacteroidetes. Unfortunately, this study failed to establish a definitive link between the increase in SCFAs and alterations in certain specific microorganisms. Future research using germ-free animals could help determine the metabolic capacity of these specific microorganisms more accurately. In addition, while our study provided promising results in a mouse model, the direct applicability of these findings to human health requires further validation through additional research and clinical studies.

## Data Availability

The raw data supporting the conclusions of this article will be made available by the authors on request.

## Supplementary Materials

The following supporting information can be downloaded at: https://www.mdpi.com/article/10.3390/nu16244365/s1, Table S1: Composition and energy supply of experimental diets.
