# Supplementary material for: Gut Microbiota and Metabolites Mediate Health Benefits of Oat and Oat Bran Consumption in IBD Mice
Source: Nutrients. 2024 Dec 18;16(24):4365. doi: 10.3390/nu16244365 (PMC11676952; doi:10.3390/nu16244365)
Supplement: Supplementary file 1 [file nutrients-16-04365-s001.zip › nutrients-3355610-supplementary.pdf]

Table S1 Composition and energy supply of experimental diets

Ingredient (g)	NC, MC	15% Oat	30% Oat	45% Oat	10% Oat bran	20% Oat bran	30% Ot bran
Corn starch	397.5	296.8	190.9	77.7	366.5	332.8	294.4
Maltodextrin 10	132	132	132	132	132	132	132
Sucrose	100	100	100	100	100	100	100
Casein	200	182.9	164.8	145.6	176.5	150.9	121.8
L-cystine	3	3	3	3	3	3	3
Cellulose, BW200	50	50	50	50	50	50	50
Soybean oil	70	60	50	38.4	67.2	64.1	60.6
Mineral mix S10022G	35	35	35	35	35	35	35
Vitamin mix V10037	10	10	10	10	10	10	10
Choline bitartrate	2.5	2.5	2.5	2.5	2.5	2.5	2.5
Oat	0	153	314	486	0	0	0
Oat bran	0	0	0	0	104	217	346
Total	1000	1025.2	1051.8	1080.1	1046.7	1097.4	1155.4
Carbohydrate (kcal %)	63.9	63.9	63.9	63.9	63.9	63.9	63.9
Protein (kcal %)	20.3	20.3	20.3	20.3	20.3	20.3	20.3
Fat (kcal %)	15.8	15.8	15.8	15.8	15.8	15.8	15.8
Total	100	100	100	100	100	100	100
